# COVID-19 vaccination and antibody response in healthcare workers: a longitudinal serological study following the 2023–2024 COVID-19 vaccination campaign

**DOI:** 10.1186/s12879-026-13571-5

**Published:** 2026-05-19

**Authors:** João Almeida Santos, Camila Henriques, Palmira Amaral, Raquel Guiomar, Ausenda Machado, Vânia Gaio

**Affiliations:** 1 Department of Epidemiology, National Institute of Health Doctor Ricardo Jorge, Avenida Padre Cruz, Lisboa, 1649-016 Portugal; 2 Department of Infectious Diseases, National Institute of Health Doctor Ricardo Jorge, Avenida Padre Cruz, Lisboa, 1649-016 Portugal; 3 Centro Hospitalar e Universitário de Tondela Viseu (Unidade Local de Saúde Viseu Dão Lafões), Avenida General Humberto Delgado, Tondela, 3460-525 Portugal; 4 Public Health Research Centre, Comprehensive Health Research Center, NOVA National School of Public Health, NOVA University Lisbon, Campo Mártires da Pátria, Lisboa, 1169-056 Portugal

**Keywords:** Healthcare workers, SARS-CoV-2 antibodies, COVID-19 vaccination, SARS-CoV-2 anti-spike receptor-binding domain, SARS-CoV-2 anti-nucleocapsid

## Abstract

**Background:**

Healthcare workers (HCWs) are essential frontline responders to public health emergencies and are one of the risk groups targeted by annual vaccination campaigns. Serological studies are valuable for high-risk groups such as HCWs, as they contribute to assess vulnerability, monitor infection control measures, and guide vaccination strategies in high-risk settings. This study aimed to assess humoral response at baseline, 3 and 6 months after the 2023–2024 COVID-19 vaccination campaign in HCWs, and to identify demographic and clinical factors associated with variations in antibody levels over time.

**Methods:**

Prospective cohort study of vaccinated HCWs at a central hospital in Portugal (September 2023–May 2024). Serial serological tests to assess anti-spike receptor-binding domain (anti-RBD/S) and anti-nucleocapsid protein (anti-N) IgG antibodies were used to monitor the immune response and SARS-CoV-2 infection history, respectively. Wilcoxon signed-rank tests were used to assess changes in antibody levels over time, and linear regression models were used to identify factors associated with variations in SARS-CoV-2 anti-RBD/S IgG concentrations, on the basis of available demographic and clinical data.

**Results:**

In a cohort of 166 HCWs who received the 2023–2024 COVID-19 booster vaccine, anti-RBD/S IgG antibody levels significantly increased at 3 months post-vaccination (16 007.4 vs. 30 572.9 AU/mL) before declining by 6 months (18 327.3 AU/mL), nearing baseline levels. Previous infection (β = 1.92, 95% CI: 1.33-2.77) and older age (β = 2.65, 95%CI: 1.64‐4.29) were associated with higher antibody concentrations at baseline, whereas smoking was linked to lower antibody levels at 6 months (β = 0.32, 95%CI: 0.11–0.88). Other factors, such as sex and chronic conditions, had no consistent significant impact over time.

**Conclusions:**

Although SARS-CoV-2 anti-RBD/S IgG antibody concentrations declined significantly six months after the 2023–2024 COVID-19 booster vaccination, they remained at relatively high concentrations over the follow-up period. This study provides new insight into these dynamics in a highly vaccinated and exposed HCWs cohort during a later post-pandemic phase, highlighting the influence of prior infection, age, and smoking on antibody persistence and reinforces the relevance of ongoing immune monitoring of this risk group to guide tailored control strategies. However, vaccine effectiveness studies in highly exposed and vaccinated populations, such as HCWs, are needed to better inform the role of antibody monitoring in this context, especially given the ongoing trend of annually updated vaccines.

**Clinical trial:**

Not applicable.

**Supplementary Information:**

The online version contains supplementary material available at 10.1186/s12879-026-13571-5.

## Background

Healthcare workers (HCWs) play a crucial role as the first line of response to public health threats. Owing to their occupational exposure, HCWs are considered a high-risk group for contracting SARS-CoV-2 infection and can pose a risk to vulnerable patients and other health professionals as potential sources of transmission [1, 2].

Seroprevalence studies offer valuable insights into the proportion of individuals who have experienced recent or past infections, while also enabling the assessment of immunity induced by vaccination. These studies are important not only at the population level but also in key population groups, such as residents of nursing homes and HCWs [3]. A meta-analysis conducted by Diznamarira et al. (2021), that included 47 studies conducted in America, Europe and Asia between 2020 and 2021, reported a random-effects adjusted pooled prevalence of SARS-CoV-2 infection of 7% (95% CI: 3%-17%) among studies that used antibody testing, and 11% (95% CI: 7–16%) among those that used PCR testing [4]. Similarly, another meta-analysis by Bansal et al. (2025) that included 63 studies conducted in America, Europe, Africa, Oceania and Asia between 2019 and 2021, reported a SARS-CoV-2 infection rate of 11% (95% CI: 9–13%), with SARS CoV-2 infection defined as laboratory-confirmed cases diagnosed through either PCR or serological testing [5]. These studies show that the burden of COVID-19 among HCWs is quite significant and therefore poses a constant global health concern, as infections disrupt health services and further strain an already limited health workforce. Additionally, the results of cohort studies conducted in the USA, Indonesia and Bangladesh indicated that after 6 months, the seropositivity of IgG anti-SARS-CoV-2 spike receptor-binding domain and neutralizing antibodies decreased significantly, although it remained detectable [6–8]. Therefore, seroprevalence studies among HCWs, regardless of symptom history, are useful, as they provide insights into the potential true extent of exposure and help assess the level of immunity among this high-risk group. Especially because serology, unlike real-time reverse transcription PCR (RT-PCR) assays, which detect SARS-CoV-2 only within the first 2–3 weeks of infection, detects the IgG-specific antibody response to SARS-CoV-2 antigens that persist for a significantly longer duration [1]. This information could help guide infection control policies and procedures, prioritize vaccination and protective measures, and ensure workforce readiness to maintain the continuity of healthcare services during seasonal epidemics [4, 5]. However, several factors have been associated with changes in the antibody response. Compared with older HCWs, younger HCWs appear to develop a higher humoral immune response to SARS-CoV-2, with a slower decline in antibody concentration [9–11]. Additionally, several studies have shown that female HCWs tend to exhibit a stronger antibody response to SARS-CoV-2 compared to their male counterparts [9, 10, 12]. In contrast, smoking and the presence of chronic conditions among HCWs have been associated with a negative impact on the antibody response to SARS-CoV-2, reducing the response and leading to a greater decline in the antibody concentration [11, 13–15].

In Portugal, the COVID-19 vaccination plan, which began on 27 December 2020, was designed to prioritize the most vulnerable individuals with the aim of preventing severe illness, hospitalization, and death, while mitigating the burden of SARS-CoV-2 on the healthcare system [16]. As they are considered a risk group, consecutive booster vaccination campaigns against COVID-19 have systematically prioritized HCWs. Following recommendation, the 2023–2024 COVID-19 vaccination campaign began on September 29, 2023, and targeted high-risk groups such as nursing home residents/professionals, the ≥ 60 years individuals, people with identified risk pathologies, pregnant women and HCWs [16]. Among the 1 992 260 individuals vaccinated against COVID-19 by April 30, 2024, individuals aged ≥ 60 years accounted for the majority (84.7%) of all vaccines administered, with vaccination coverage in this group reaching 56.1%. HCWs represented 2.6% of the total vaccinated population, with 52 101 professionals vaccinated [17].

Since 2020, Portugal implemented a prospective cohort study targeting HCWs at the hospital setting, with the objective of evaluating vaccine effectiveness (VE) against laboratory confirmed (RT-PCR) SARS-CoV-2 infection. Within this VE study, serological antibody monitoring was conducted [18]. Since 2021, the European Centre for Disease Prevention and Control (ECDC) initiated the Vaccine Effectiveness, Burden and Impact Studies (VEBIS), a platform for multicentre European vaccine effectiveness (VE) and other studies in different settings [19]. One of the components of this platform aimed at assessing the effectiveness of the COVID-19 vaccines among HCWs (VEBIS HCW study) [20], as HCW cohorts offer a unique opportunity to study COVID-19 vaccine effects in a well-defined, easily monitored group of mostly healthy working-age adults. Portugal has been participating in the VEBIS HCW study since its beginning.

Thus, recognizing the importance of monitoring COVID-19 vaccination in HCWs, the present study aimed to assess IgG antibodies specific to the SARS-CoV-2 spike receptor-binding domain and nucleocapsid protein at baseline, 3 and 6 months after the COVID-19 2023–2024 vaccination program in HCWs, and to identify demographic and clinical factors associated with variations in antibody levels over time.

## Methods

### Study design, setting and Participants

All procedures implemented in this cohort study were based on the guidance document “Cohort study to measure COVID- 19 VE among health workers in the WHO European Region” [21]. The VEBIS HCW study is a prospective cohort study, and the HCWs included in this analysis were drawn from the cohort participating in the COVID-19 vaccine effectiveness study conducted from September 2023 to May 2024 at Centro Hospitalar Tondela-Viseu (CHTV), a central hospital located in the central region of Portugal.

HCWs from all categories of staff with no vaccine contraindication, who did not present with special recommendations for vaccination (i.e., immunocompromised participants with a three-dose primary course), who did not present an immunization event (vaccination or infection) in the previous three months and who provided informed consent were included.

HCWs were invited to participate in the study regardless of their intention to be vaccinated or their vaccination status. All HCWs at the CHTV were invited to participate via institutional email via occupational medicine services before the beginning of the COVID-19 2023–2024 vaccination program (September 29, 2023). At recruitment, a nasopharyngeal or saliva sample for RT-PCR testing for SARS-CoV-2 infection was collected, and demographic, clinical (vaccination history, prior infection with SARS-CoV-2) and in-hospital and community-related behavioural data were gathered through an online questionnaire. At follow-up, the participating HCWs provided weekly samples to test for SARS-CoV-2 infection using RT-PCR and completed a weekly questionnaire to record changes in vaccination status and professional and community risk for infection. All questionnaires were based on the “Generic Protocol for ECDC studies of COVID-19 vaccine effectiveness against confirmed SARS-CoV-2 infection using Healthcare Worker Cohorts” [20]. Blood samples were collected at three time points throughout the study: pre-vaccination or baseline (September/October 2023), three months (January/February 2024), and six months (April/May 2024) post-COVID-19 2023–2024 vaccination.

Among the participants who consented to participate in the VEBIS HCW study, all HCWs who were vaccinated in the 2023–2024 COVID-19 vaccination campaign and who underwent pre-vaccination serology for IgG antibodies specific to SARS-CoV-2 spike receptor-binding domain and to SARS-CoV-2 nucleocapsid protein were selected for the present study.

### Serological tests

The quantitative determination of protein-specific IgG antibodies against the SARS-CoV-2 spike receptor-binding domain (anti-RBD/S IgG) and the qualitative detection of nucleocapsid protein-specific IgG antibodies (anti-N IgG) were performed at the National Reference Laboratory for Influenza Virus and Other Respiratory Viruses at the National Institute of Health Doctor Ricardo Jorge, via chemiluminescence microparticle immunoassays (CMIA) in the ARCHITECT i1000SR system, according to the manufacturer’s instructions.

Anti-RBD/S IgG quantitative detection was performed in serum samples through the SARS-CoV-2 IgG II Quant assay (Abbott Diagnostics, Abbott Park, IL, USA). In accordance with the manufacturer’s instructions, the serum samples were considered positive for anti-RBD/S IgG when the results were ≥ 50 AU/mL. Anti-N IgG qualitative detection was performed in serum samples via the SARS-CoV-2 IgG assay (Abbott Diagnostics, Abbott Park, IL, USA). According to manufacturer’s instructions, serum samples were considered positive for IgG anti-N if the ratio of the sample’s optical density to the calibrator’s optical density (index S/C) was ≥ 1.40.

Anti-N IgG was used as a proxy for previous SARS-CoV-2 infection (anti-N IgG < 1.4 = negative, no previous infection; anti-N IgG ≥ 1.4 = positive, evidence of previous infection).

### Statistical analyses

Sociodemographic and health characteristics of the participants were described as relative frequencies for categorical variables, and mean and standard deviations for numerical variables. Chi-square tests and Kruskal-Wallis tests were conducted accordingly to assess whether the participants’ distributions differed significantly across the three rounds of serological testing.

The number of COVID-19 cases that occurred in Portugal between September 2023 and May 2024 by ISO week/year were retrieved from the Directorate General of Health (Direção-Geral da Saúde), the Portuguese institution responsible for public health surveillance, policy implementation, and epidemiological reporting [22]. These data were plotted against the proportion of previous/new SARS-CoV-2 infections in the studied population.

Data on the quantification of anti-RBD/S IgG are represented as an estimated geometric mean with a 95% confidence interval (95% CI). Wilcoxon’s signed-rank test was applied to assess significant differences in anti-RBD/S IgG geometric mean concentrations across the different time points of observation: between baseline and 3 months, between 3 and 6 months, and between baseline and 6 months post-vaccination. This analysis was stratified by sex, age group, smoking status, presence of chronic conditions and history of previous infection.

Linear regression models were used to identify factors associated with variations in anti-RBD/S IgG concentrations over time, with variable selection on the basis of current scientific evidence and data availability. A multiple linear regression model was fitted at each time point, including sex (male, female), age group (25–44, 45–54, 55 + years), smoking status (nonsmoker, smoker), chronic conditions (no, yes), and previous infection status (no, yes) as covariates. All tests were 2-sided, with statistical significance set at α = 0.05. Analyses were performed via Stata software, version 15.1 (StataCorp.2017. Stata Statistical Software, StataCorp, College Station, TX, USA).

## Results

**Fig. 1:**
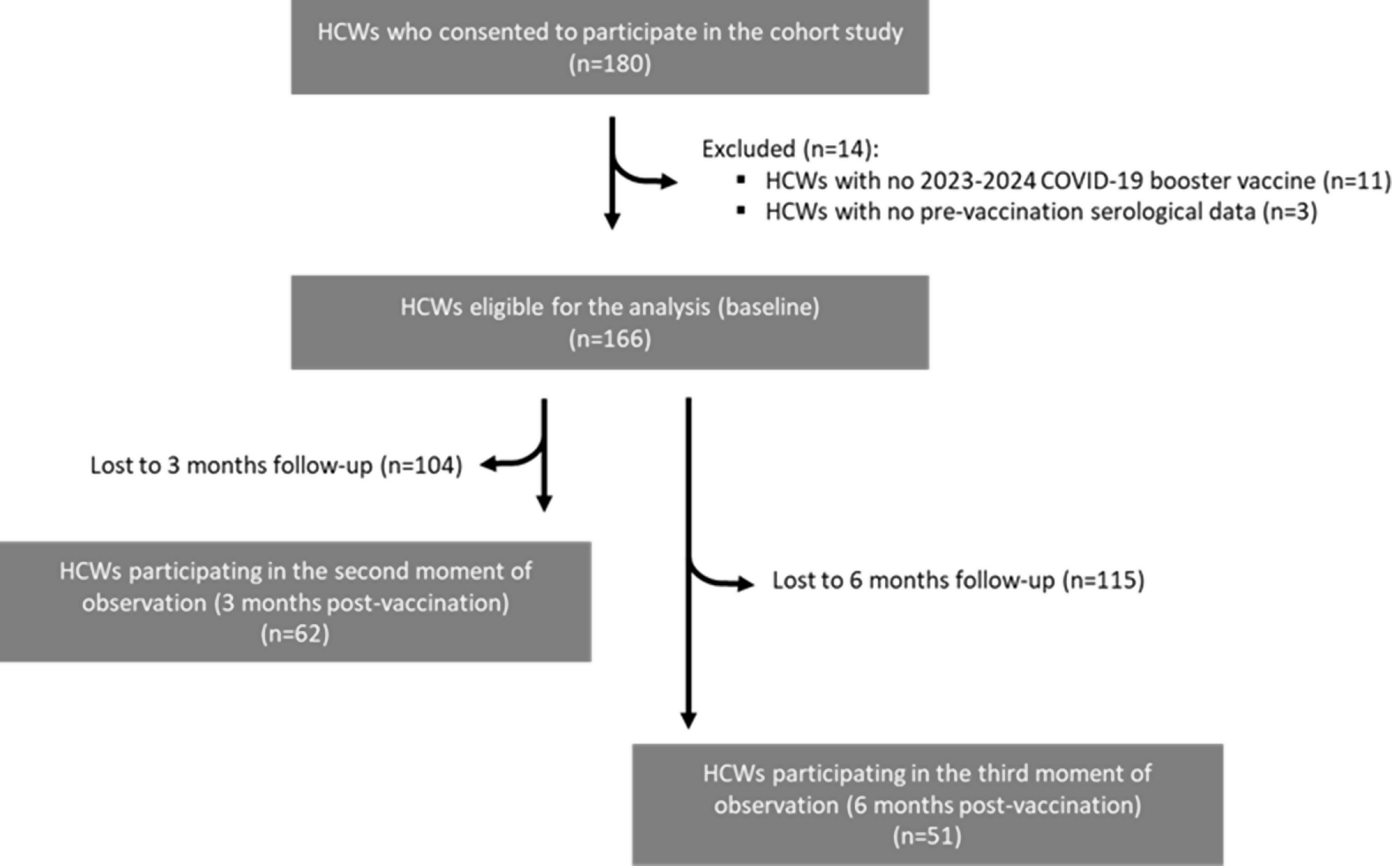
Flowchart of HCWs participation at baseline, and at the 3-month, and 6-month follow-ups

Among the 180 HCWs who consented to participate in the VEBIS HCW cohort study, 11 (6.1%) were excluded for not receiving the 2023–2024 COVID-19 booster vaccine, and 3 (1.6%) were excluded because of the absence of pre-vaccination serological data. Consequently, the eligible study population consisted of 166 participants (92.2%) – Fig. 1. A comparison of sex and age distributions between hospital HCWs and those included in the study is presented in Supplementary Table 1.

The majority of HCWs were female (126/166; 75.9%), with a mean age of 47.9 years (sd ± 9.0; range: 29–71 years), 14.3% (11/77) reported being smokers, and 54.6% (42/77) reported having at least one chronic condition – Table 1. All participants had completed the primary COVID-19 vaccination series, with the majority (133/166; 80.1%) having received three or more additional booster doses.

**Table 1:** Participants’ sociodemographic and health characteristics at baseline, 3 months and 6 months post-vaccination

Variables	Baseline		3 months		6 months		*p*-value
	*n*	%	*n*	%	*n*	%	
**Total**	166		62	37.3	51	30.7	
**Sex (** ***n*** ** = 166)**							
Female	126	75.9	48	77.4	38	74.5	0.936^*^
Male	40	24.1	14	22.6	13	25.5	
**Age, mean [range] years**	47.9 [29–71]		49.8 [35–71]		50.7 [36–71]		0.130^†^
**Age group (** ***n*** ** = 166)**							
25–44	65	39.2	20	32.3	14	27.4	0.369^*^
45–54	58	34.9	20	32.3	18	35.3	
55+	43	25.9	22	35.5	19	37.3	
**Smoking (** ***n*** ** = 77)**							
Nonsmoker	66	85.7	46	95.8	38	92.7	0.149^*^
Smoker	11	14.3	2	4.2	3	7.3	
**Chronic condition (*n*** **= 77)**							
No	35	45.5	24	50.0	20	48.8	0.871^*^
Yes	42	54.5	24	50.0	21	51.2	
**Previous infection (*n*** **= 166)**							
No	85	51.2	35	56.4	35	68.6	0.089^*^
Yes	81	48.8	27	43.6	16	31.4	

^*^Chi-Square test ^†^Kruskal-Wallis test

**Fig. 2:**
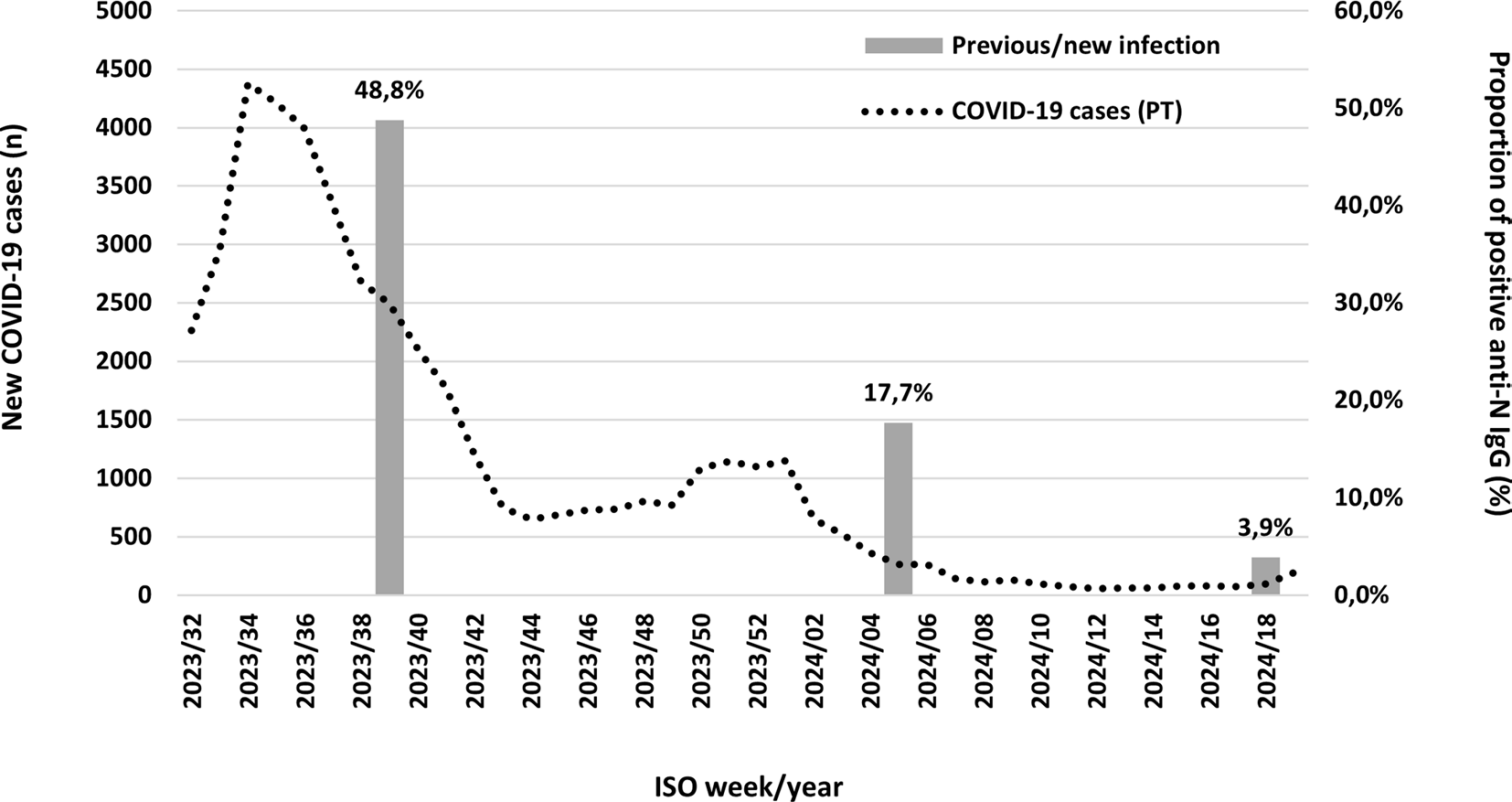
Number of new COVID-19 cases in Portugal (dotted line) by ISO week/year between September 2023 and May 2024 [22], and the proportions of previous infections at baseline, and new infections at 3 and 6 months post-vaccination (bars)

Although the number of HCWs at the three time points of observation varied, all demographic and health status indicators (sex, age, smoking status, presence of chronic conditions and previous infection status) remained similar across the three time points, with no significant differences detected. Nevertheless, from the 81 (48.8%) cases of previous infection identified at baseline, the detection of new cases declined markedly to 11 (17.7%) at 3 months and further to 2 (3.9%) at 6 months. This variation across the different time points was statistically significant (*p* < 0.001). Figure 2 shows the number of COVID-19 cases that occurred in Portugal between September 2023 and May 2024 (dotted line) against the proportion of cases of previous infection at baseline and new cases of infection three and six months after vaccination. Supplementary Tables 2 and 3 compare participants and non-participants at 3 and 6 months following COVID-19 vaccination. At 3 months, only smoking status differed significantly between the two groups, whereas at 6 months, a significant difference was observed only for age.

**Fig. 3:**
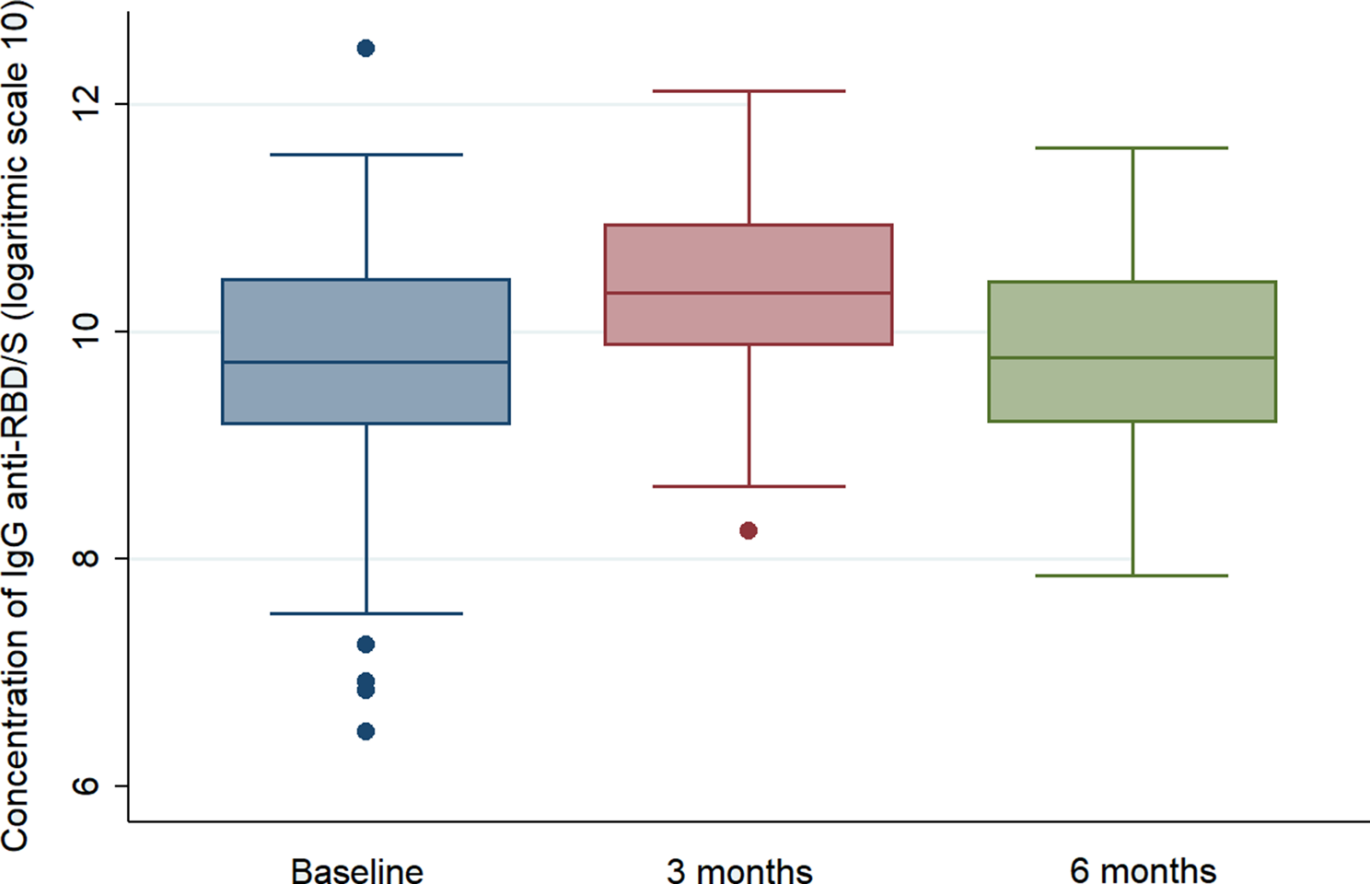
Anti-RBD/S IgG concentration (logarithmic_10_ scale) reported in the boxplots (and outliers) for participants at the three different moments of observation

Regarding the anti-RBD/S IgG concentration, the geometric mean concentration of anti-RBD/S IgG was lowest at baseline (16 007.4 AU/mL) and peaked 3 months post-vaccination (30 572.9 AU/mL), suggesting an effective immune response to the 2023–2024 COVID-19 booster vaccination - Fig. 3. However, by 6 months post-vaccination, the geometric mean of anti-RBD/S IgG levels had declined to 18 327.3 AU/mL, approaching baseline levels, although the minimum value at 6 months remained substantially greater than the one at baseline (2 586.4 vs. 649.7 AU/mL) – Table 2. The increase from baseline to 3 months and the subsequent decline between 3 and 6 months were both statistically significant (*p* < 0.001), yet by 6 months, the anti-RBD/S IgG concentrations were no longer significantly different from those at baseline (*p* = 0.07). Boxplots reporting anti-RBD/S IgG concentrations (logarithmic_10_ scale) by sex, age, smoking status, and chronic conditions status are presented in Supplementary Figs. 1 to 4.

**Table 2:** Geometric mean of anti-RBD/S IgG concentration at baseline, 3 and 6 months, overall and stratified by sociodemographic and health characteristics, with pairwise comparisons

Variable		Baseline (AU/mL)	3 months (AU/mL)	6 months (AU/mL)	Baseline vs. 3 months (*p*-value*)	3 months vs. 6 months (*p*-value*)	Baseline vs. 6 months (*p*-value*)
**Total**	**Geometric Mean**	16 007.4	30 572.9	18 327.3	**< 0.001**	**< 0.001**	0.07
	**[95% CI]**	13 800.1–18 567.7	24 736.8–37 785.8	14 209.6–23 638.1			
	**Min-Max**	649.7–266 139.0	3 802.3–181 952.9	2 586.4–110 442.7			
**Sex**							
Female	**Geometric Mean**	15 974.3	31 660.4	17 519.6	**< 0.001**	**< 0.001**	0.09
	**[95% CI]**	13 516.5–18 879.1	25 074.8–39 975.7	13 516.5–18 879.1			
Male	**Geometric Mean**	16 111.9	27 119.9	20 907.9	0.140	**0.034**	0.422
	**[95% CI]**	11 541.9–22 491.3	15 622.6–47 078.6	11 128.3–39 281.9			
**Age group**							
25–44	**Geometric Mean**	12 608.5	28 921.9	13 407.9	**< 0.001**	**0.002**	0.246
	**[95% CI]**	10 189.4–15 601.8	21 031.2–39 773.4	8 944.4–20 099.0			
45–54	**Geometric Mean**	17 945.4	30 116.4	19 847.6	**0.023**	**0.005**	0.327
	**[95% CI]**	14 284.4–22 544.8	18 815.1–48 205.8	12 181.5–32 338.3			
55+	**Geometric Mean**	19 681.9	32 598.1	21 395.5	**0.019**	**0.01**	0.334
	**[95% CI]**	13 702.4–28 270.7	22 615 − 46 987.9	13 539.9–33 808.7			
**Smoking**							
Nonsmoker	**Geometric Mean**	15 504.3	34 631.8	19 459.1	**< 0.001**	**< 0.001**	0.162
	**[95% CI]**	12 339.4–19 480.9	27 614.7–43 431.9	15 061.4–25 140.9			
Smoker	**Geometric Mean**	12 018.1	11 266.5	5 063.8	0.655	0.317	0.593
	**[95% CI]**	7 156.2–20 183.2	0.01–1.111 × 10¹⁰	943.7–27 171.0			
**Chronic condition**							
No	**Geometric Mean**	13 312.397	35 517.3	17 790.0	**< 0.001**	**< 0.001**	**0.025**
	**[95% CI]**	9 735.9–18 202.7	25 923.2–48 662.1	12 818.8–24 689.1			
Yes	**Geometric Mean**	16 468.2	30 751.7	17 486.2	**0.01**	**0.002**	0.543
	**[95% CI]**	12 422.0–21 832.2	21 188.1–44 631.9	11 206.2–27 285.6			
**Previous infection**							
No	**Geometric Mean**	12 412.1	23 675.6	16 251.6	**< 0.001**	**< 0.001**	0.088
	**[95% CI]**	9 962.7–15 463.9	17 732.0–31 611.5	11 840.9–22 305.2			
Yes	**Geometric Mean**	20 905.0	42 586.3	23 839.0	**< 0.001**	**< 0.001**	0.228
	**[95% CI]**	17 370.0–25 159.3	32 158.1–56 396.2	15 286.1–37 177.2			

CI, Confidence interval; *Wilcoxon Signed-Rank Test (*p* < 0.05 was considered significant)

Stratified analysis revealed differences in the geometric mean concentrations of anti-RBD/S IgG over time by sex, age group, smoking status, and presence of chronic conditions. Geometric mean anti-RBD/S IgG concentrations increased from baseline to 3 months among female, nonsmoking participants, across all age groups, and regardless of chronic conditions or previous infection status, and this increase was statistically significant (*p* < 0.05). In contrast, the increase observed among male participants was not statistically significant (*p* = 0.140). Smokers were the only HCWs who presented a decrease in the geometric mean anti-RBD/S IgG concentration from baseline to 3 months, although this decrease was not statistically significant (*p* = 0.655). Between 3 and 6 months, a decrease in concentrations was observed across sex, age groups, smoking status, presence of chronic conditions, and previous infection status. This decrease was statistically significant for all categories, except for smokers (*p* = 0.317). When comparing baseline to 6 months, only participants without chronic conditions showed a statistically significant difference in geometric mean anti-RBD/S IgG concentrations (*p* = 0.025).

Table 3 shows the results of the linear regression analysis of log-transformed anti-RBD/S IgG concentrations at baseline, 3 months, and 6 months post-vaccination. At baseline, previous infection was associated with higher anti-RBD/S concentrations (β = 1.92, 95% CI: 1.33-2.77). Age 45–54 years (β = 1.64, 95% CI: 1.06‐2.52) and ≥ 55 years (β = 2.65, 95% CI: 1.64‐4.29) were also associated with higher concentrations, when compared to 25–44 years age group. At 3 months, detection of anti-N remained associated with higher anti-RBD/S concentrations (β = 1.95, 95% CI: 1.21–3.13). No significant associations were found for age, sex, or smoking. A borderline significant negative association was found for the presence of chronic conditions (β = 0.63, 95% CI: 0.40–1.00). At 6 months, smoking was significantly associated with lower anti-RBD/S IgG concentrations (β = 0.32, 95% CI: 0.11–0.88). No significant associations were found for sex, age group, chronic conditions, or previous infection.

**Table 3:** Linear regression model on log-transformed anti-RBD/S IgG concentration at baseline, 3 months, and 6 months post-vaccination

Variables	Baseline		3 Months		6 Months	
	β	*p*-value [95% CI]	β	*p*-value [95% CI]	β	*p*-value [95% CI]
**Sex**						
Female	*		*		*	
Male	1.45	0.097 [0.93–2.24]	1.48	0.155 [0.93–2.24]	1.22	0.482 [0.69–2.15]
**Age group**						
25–44	*		*		*	
45–54	1.64	**0.026** **[1.06–2.52]**	1.16	0.576 [0.68–1.97]	1.16	0.625 [0.63–2.13]
55+	2.65	**< 0.001** **[1.64–4.29]**	1.60	0.081 [0.94–2.72]	1.80	0.075 [0.94–3.43]
**Smoking**						
Nonsmoker	*		*		*	
Smoker	0.99	0.957 [0.58–1.68]	0.44	0.138 [0.15–1.32]	0.32	**0.028** **[0.11–0.88]**
**Chronic condition**						
No	*		*		*	
Yes	1.09	0.655 [0.75–1.56]	0.63	0.050 [0.40–1.00]	0.91	0.743 [0.52–1.59]
**Previous infection**						
No	*		*		*	
Yes	1.92	**0.001** **[1.33–2.77]**	1.95	**0.007** **[1.21–3.13]**	1.57	0.131 [0.87–28.4]

*Constant, reference category; CI, Confidence interval

## Discussion

This prospective cohort study evaluated the dynamics of the SARS-CoV-2 anti-RBD/S IgG antibody response following the 2023–2024 COVID-19 booster vaccination among HCWs in a hospital setting in Portugal.

Our findings indicate significant changes in anti-RBD/S IgG concentrations over the six-month follow-up period after the 2023–2024 COVID-19 booster vaccination. The geometric mean concentration of anti-RBD/S IgG antibodies increased significantly three months after vaccination. However, by six months, antibody levels had declined significantly, returning to near pre-vaccination levels. This observation aligns with the existing literature on the waning of humoral immunity following COVID-19 vaccination [6–8, 11, 23]. This transient increase and subsequent decline in antibody levels reaffirmed the temporary nature of post-vaccination immunity and highlights the importance of continued monitoring to inform the need for the implementation or reinforcement of protective measures, such as mask wearing, physical distancing, contact-tracing investigations or booster COVID-19 vaccine campaigns - particularly for high-risk HCWs who face sustained exposure risk [24–26]. Despite the decline, the minimum antibody levels remained higher than those at baseline, indicating that the participants retained immune memory, as previously described [27].

It is important to emphasize that although the concentration of anti-RBD/S IgG antibodies decreased over time, baseline levels were already high. While it is widely accepted that COVID-19 vaccine effectiveness wanes over time, our data show that antibody levels, despite declining, remain at high concentrations, possibly reflecting the cumulative impact of multiple vaccination campaigns and natural infections since the onset of the epidemic. These findings highlight the importance of continued long-term follow-up and maintaining cohorts such as the one in this study to monitor immune dynamics effectively following vaccination and reinfection.

In the stratified analysis, participants with prior SARS-CoV-2 infection (anti-N positive) consistently presented higher geometric mean concentrations of anti-RBD/S IgG compared to those without prior exposure (anti-N negative) across all time points. The associations between prior SARS-CoV-2 infection and higher anti-RBD/S IgG concentrations were significant at baseline (1.92, 95% CI: 1.33–2.77) and at 3 months (1.95, 95% CI: 1.21–3.13) post-vaccination, suggesting an association between prior infection and higher antibody levels. At 6 months, the marked decline in new infections (2/51), may explain the inability to establish an association between prior SARS-CoV-2 infection and higher anti-RBD/S IgG concentrations. Such results align with studies highlighting the enhanced immune response in individuals with hybrid immunity (i.e., vaccination plus previous infection), who develop a more robust and broader immune response, offering extended protection against SARS-CoV-2, including emerging variants, which has been observed for up to six months [28–30]. These findings emphasize the role of past infection in shaping vaccine-induced immune responses, reflecting the additive effect of hybrid immunity, whereby infection-induced immunity combined with vaccination is associated with higher antibody concentrations [27, 28, 31, 32]. However, even among participants with hybrid immunity, antibody levels decreased significantly by 6 months, and the association with prior infection was not statistically significant (1.57, 95% CI 0.87–28.4). These results support the evidence that hybrid immunity does not prevent the waning of antibody concentrations over time [31, 33, 34].

Interestingly, the number of new HCWs with prior infection declined over time [81 (baseline) vs. 11 (3 months) vs. 2 (6 months)], suggesting reduced natural infection rates during the study period, which is consistent with Portugal’s declining number of COVID-19 cases during the time frame of the study [22]. The first two observation periods (baseline and 3 months post-vaccination) occurred following a surge in COVID-19 cases, which likely contributed to the higher number of serologically detected infections. In contrast, during the third observation period, the incidence of COVID-19 was low, which was reflected in the reduced number of new infections recorded between 3- and 6-months post-vaccination. Thus, monitoring anti-SARS-CoV-2 antibody levels among HCWs could serve as a valuable tool for understanding transmission dynamics within healthcare settings, as it may provide early indications of rising infection rates in the broader population and help assess the ongoing effectiveness of preventive measures.

With respect to age, older HCWs (45–54 and 55+) were significantly associated with higher geometric mean concentrations of anti-RBD/S IgG at baseline than were those in the 25–44 age group; however, this association was not observed at 3 months or 6 months post-vaccination. Nevertheless, a pattern toward higher antibody concentrations persisted among HCWs aged 55 years and older, with the association approaching statistical significance. These findings may reflect greater cumulative exposure among older HCWs, potentially due to prolonged occupational exposure to SARS-CoV-2 or additional vaccine doses. However, previous studies have shown contrasting results, suggesting that younger HCWs may exhibit more robust immunological responses and, consequently, higher concentrations of anti-RBD/S antibodies [9, 10, 14]. Even so, several of these studies primarily assessed immune responses following initial vaccination or first-wave SARS-CoV-2 infections, during which younger individuals often demonstrated higher primary immune responses, potentially reflecting differences in innate immune function. In contrast, our study, which was conducted more recently, may reflect the cumulative effect of prolonged exposure among older HCWs. This increased exposure likely resulted in a higher incidence of asymptomatic or mild infections, contributing to immune priming, in addition to more frequent administration of booster vaccinations. Nonetheless, by six months post-vaccination, age was not significantly associated with anti-RBD/S IgG concentrations, suggesting similar rates of antibody waning across age groups.

In terms of smoking status, smokers were the only HCWs subgroup that did not exhibit an increase in anti-RBD/S IgG concentrations at 3 months post-vaccination. Instead, their antibody concentrations demonstrated a steady decline from baseline through 6 months post-vaccination. Furthermore, smoking was significantly associated with lower anti-RBD/S IgG geometric mean concentrations at 6 months (0.028; 95% CI: 0.11–0.88). These findings align with previous research suggesting that smoking impairs both innate and adaptive immune functions, thereby negatively affecting the antibody response [14, 35, 36].

Sex and having at least one chronic condition revealed no consistent or significant associations with antibody concentrations over time. Our findings are consistent with those reported in several studies conducted among HCWs [36–38], which similarly reported no significant associations between sex and anti-RBD/S IgG concentrations. The absence of a significant association may be partly attributable to limitations in sample size, particularly the smaller number of male HCWs, as the number of female participants was approximately three times greater at all time points. No significant differences were observed between HCWs with and without chronic conditions, which contrasts with studies reporting reduced immunogenicity in immunocompromised individuals [10, 15]. This finding may be explained by the fact that our cohort consists of active health professionals who are likely to benefit from effective monitoring and management of their chronic conditions through occupational health services, potentially mitigating the impact of chronic conditions on the humoral response.

This study it is not without limitations. The loss of participants to follow-up over time (only 30.7% of participants remained at six months) may have introduced attrition bias and the observed antibody trajectories may not fully represent the original cohort. Although most baseline characteristics remained comparable across time points, some differences between participants and non-participants were observed (notably age at 6 months and smoking status at 3 months), which may have influenced estimates of antibody dynamics. Also, mixed-effects models, which can better account for within-subject correlation in longitudinal data, were not applied. Although such models could provide a more robust analytical framework, their use was not feasible due to the reduced and unbalanced sample across time points. This may have limited our ability to fully capture individual-level trajectories of antibody response over time. A limitation of this study is its observational design without a control group. As such, although we describe the dynamics of humoral responses following booster vaccination, we are unable to determine the extent to which the observed antibody levels correlate with protection against SARS-CoV-2 infection, particularly in a population with high levels of prior vaccination and infection. Prior infections may also have been underreported, as anti-N antibodies may not always rise to detectable levels, especially if the reinfection is mild or rapidly controlled by existing immunity. Not all questionnaires were fully completed, resulting in fewer available responses for certain variables than the total sample size (e.g., smoking status and presence of chronic conditions). We acknowledge this as a limitation of the study, as the missing data may have reduced the completeness and robustness of the analyses involving these variables. It is also important to acknowledge the potential for selection bias. Although random selection was implemented a priori, the final cohort ultimately consisted of individuals who were more inclined to participate in a long-term follow-up study, suggesting some self-selection. However, the study has several strengths, including its prospective design, the use of validated assays for serial antibody measurements, and its focus on a key risk-group. Additionally, this study was conducted recently, in the post-pandemic period, within a highly vaccinated and highly exposed cohort, at a time when fewer studies continue to monitor the immunological dynamic of vaccine induced antibodies. The results of this study, together with ongoing monitoring, may contribute valuable insights into the effect of repeated vaccination.

## Conclusion

In conclusion, the COVID-19 booster administered in 2023–2024 was associated with a marked increase in antibody concentrations in the short term, which waned significantly by 6 months. Prior SARS-CoV-2 infection, older age, and smoking status influenced antibody levels, whereas sex and chronic conditions had limited impact. These findings suggest a potential role for monitoring antibody levels and tailoring booster strategies based on HCWs’ individual risk factors and occupational exposure. However, this approach depends on the extent to which antibody levels correlate with protection in multiple infected and/or vaccinated HCWs against currently circulating strains, particularly in the context of annually updated vaccines. Therefore, vaccine effectiveness studies in highly exposed and vaccinated populations, such as HCWs, are needed to better use antibody monitoring in this context.

## Supplementary Information

Below is the link to the electronic supplementary material.

Supplementary Material 1: An additional file is provided (Supplementary data.docx) with: (a) Supplementary Table 1. Comparison of sex and age distribution between all hospital healthcare workers (Hospital HCWs) and those included in the study (Study HCWs); (b) Supplementary Table 2. Comparison between participants and non-participants at 3 months post-COVID-19 vaccination; (c) Supplementary Table 3. Comparison between participants and non-participants at 6 months post-COVID-19 vaccination. (d) Supplementary Figure 1. Anti-RBD/S IgG concentration (logarithmic10 scale) reported in the boxplots (and outliers) for participants by sex at the three different time points of observation; (e) Supplementary Figure 2. Anti-RBD/S IgG concentration (logarithmic10 scale) reported in the boxplots (and outliers) for participants by age group at the three different time points of observation; (f) Supplementary Figure 3. Anti-RBD/S IgG concentration (logarithmic10 scale) reported in the boxplots (and outliers) for participants by smoking status at the three different time points of observation; (g) Supplementary Figure 4. Anti-RBD/S IgG concentration (logarithmic10 scale) reported in the boxplots (and outliers) for participants by chronic condition status at the three different time points of observation

## Data Availability

Data are available upon reasonable request.

## Abbreviations

95% CI: 95% Confidence Interval
Anti-N: Antibody against SARS-CoV-2 nucleocapsid protein
Anti-RBD/S: Antibody against SARS-CoV-2 spike receptor-binding domain
CMIA: Chemiluminescence microparticle immunoassays
COVID-19: Coronavirus Disease 2019
HCWs: Healthcare workers
SARS-CoV-2: Severe Acute Respiratory Syndrome Coronavirus 2
