# Supplementary material for: COVID-19 vaccination and antibody response in healthcare workers: a longitudinal serological study following the 2023–2024 COVID-19 vaccination campaign
Source: BMC Infect Dis. 2026 May 19;26:1327. doi: 10.1186/s12879-026-13571-5 (PMC13366825; doi:10.1186/s12879-026-13571-5)

# Supplementary data

| **Supplementary Table 1.** Comparison of sex and age distribution between all hospital healthcare workers (Hospital HCWs) and those included in the study (Study HCWs). | | | |
| --- | --- | --- | --- |
| **Variables** | **Hospital HCWs**  **n (%)** | **Study HCWs**  **n (%)** | **p-value^1^** |
| **Total** | 2781 | 166 |  |
| **Sex** |  |  |  |
| **Female** | 2116 (76.1) | 126 (75.9) | 0.957 |
| **Male** | 665 (23.9) | 40 (24.1) |  |
| **Age group** |  |  |  |
| **25-44** | 1336 (48.0) | 65 (39.2) | 0.070 |
| **45-54** | 796 (28.6) | 58 (34.9) |  |
| **55+** | 638 (22.9) | 43 (25.9) |  |
| **^1^** Chi-Square test; HCWs, healthcare workers. | | | |

| **Supplementary Table 2.** Comparison between participants and non-participants at 3 months post-COVID-19 vaccination. | | | | | |
| --- | --- | --- | --- | --- | --- |
| **Variables** | **Participants** | | **Non-participants** | | **p-value** |
|  | **n** | **%** | **n** | **%** |  |
| **Total** | 62 | 37.3 | 104 | 62.7 |  |
| **Sex (n=166)** |  |  |  |  |  |
| **Female** | 48 | 77.4 | 78 | 75.0 | 0.724^*^ |
| **Male** | 14 | 22.6 | 26 | 25.0 |  |
| **Age, mean [range] years** | 49.8 [35-71] |  | 46.8 [29-66] |  | 0.068^†^ |
| **Age group (n=166)** |  |  |  |  |  |
| **25-44** | 20 | 32.3 | 45 | 43.3 | 0.086^*^ |
| **45-54** | 20 | 32.3 | 38 | 36.5 |  |
| **55+** | 22 | 35.5 | 21 | 20.2 |  |
| **Smoking (n=77)** |  |  |  |  |  |
| **Nonsmoker** | 46 | 95.8 | 20 | 69.0 | 0.001^*^ |
| **Smoker** | 2 | 4.2 | 9 | 31.0 |  |
| **Chronic condition (n=77)** |  |  |  |  |  |
| **No** | 24 | 50.0 | 11 | 37.9 | 0.303^*^ |
| **Yes** | 24 | 50.0 | 18 | 62.1 |  |
| ^*^Chi-Square test ^†^Kruskal-Wallis test | | | | | |

| **Supplementary Table 3.** Comparison between participants and non-participants at 6 months post-COVID-19 vaccination. | | | | | |
| --- | --- | --- | --- | --- | --- |
| **Variables** | **Participants** | | **Non participants** | | **p-value** |
|  | **n** | **%** | **n** | **%** |  |
| **Total** | 51 | 30.7 | 115 | 69.3 |  |
| **Sex (n=166)** |  |  |  |  |  |
| **Female** | 38 | 74.5 | 88 | 76.5 | 0.780^*^ |
| **Male** | 13 | 25.5 | 27 | 23.5 |  |
| **Age, mean [range] years** | 50.7 [36-71] |  | 46.6 [29-66] |  | 0.010^†^ |
| **Age group (n=166)** |  |  |  |  |  |
| **25-44** | 14 | 27.4 | 51 | 44.4 | 0.044^*^ |
| **45-54** | 18 | 35.3 | 40 | 34.8 |  |
| **55+** | 19 | 37.3 | 24 | 20.9 |  |
| **Smoking (n=77)** |  |  |  |  |  |
| **Nonsmoker** | 38 | 92.7 | 28 | 77.8 | 0.062^*^ |
| **Smoker** | 3 | 7.3 | 8 | 22.2 |  |
| **Chronic condition (n=77)** |  |  |  |  |  |
| **No** | 20 | 48.8 | 15 | 41.7 | 0.532^*^ |
| **Yes** | 21 | 51.2 | 21 | 58.3 |  |
| ^*^Chi-Square test ^†^Kruskal-Wallis test | | | | | |

**Supplementary Figure 1.** Anti-RBD/S IgG concentration (logarithmic_10_ scale) reported in the boxplots (and outliers) for participants by sex at the three different time points of observation.


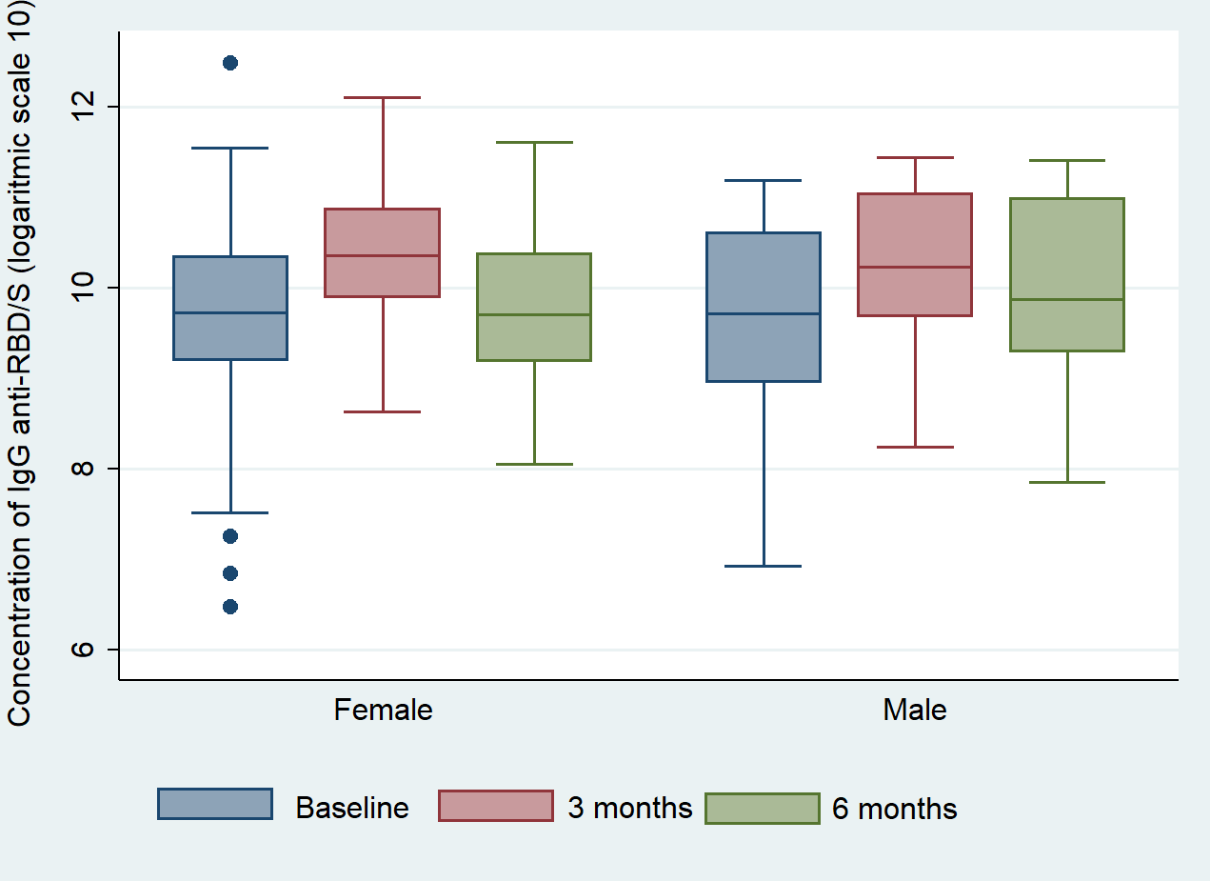

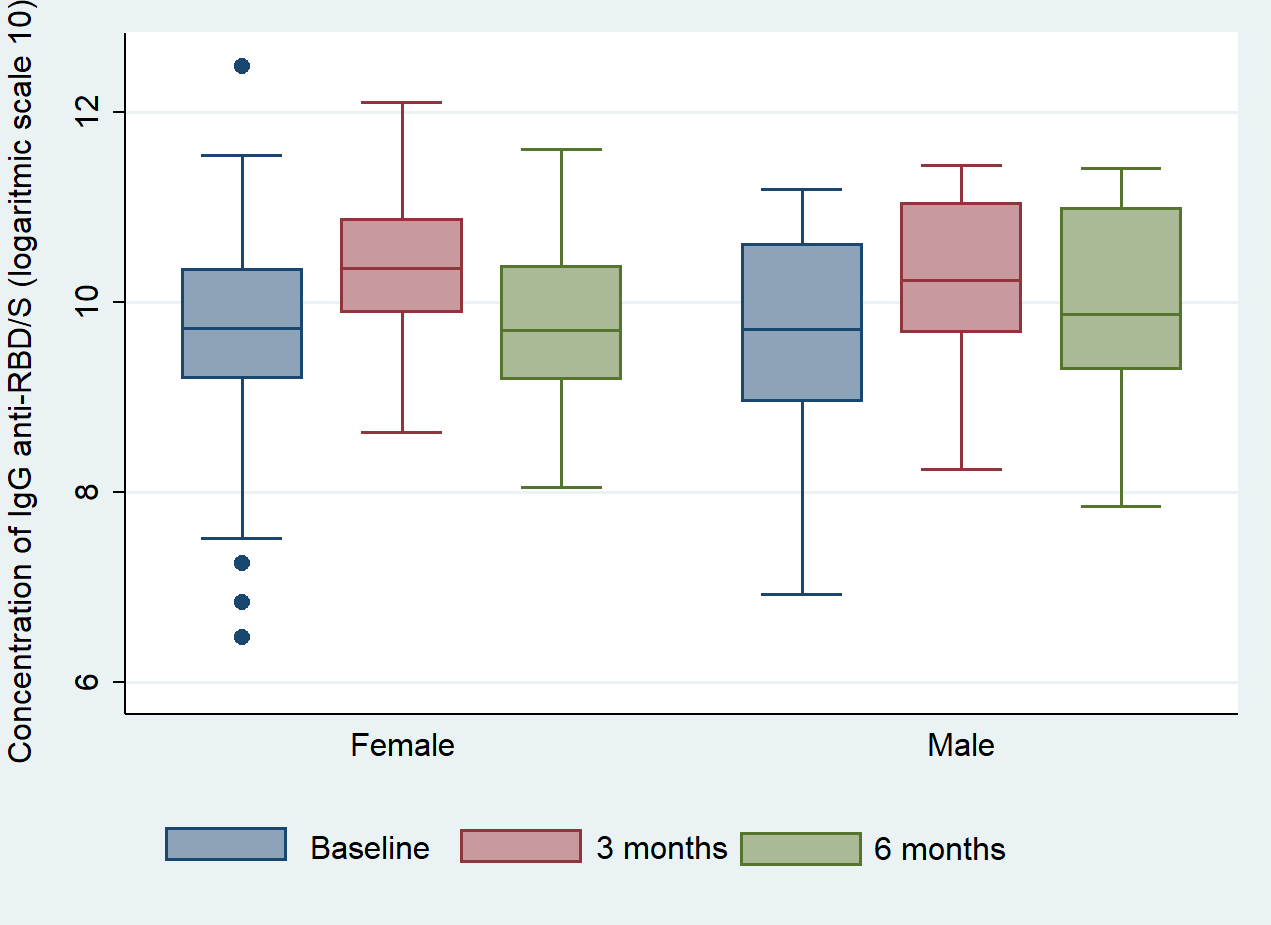


**Supplementary Figure 2.** Anti-RBD/S IgG concentration (logarithmic_10_ scale) reported in the boxplots (and outliers) for participants by age group at the three different time points of observation.


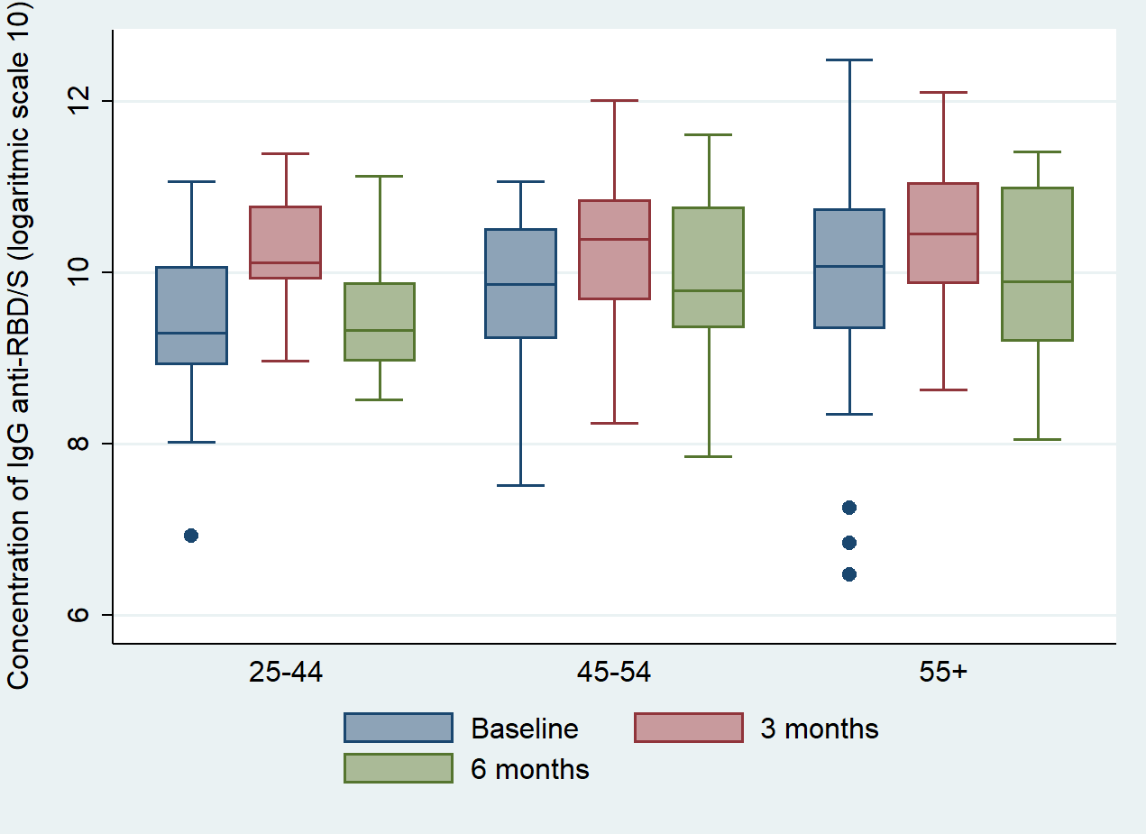

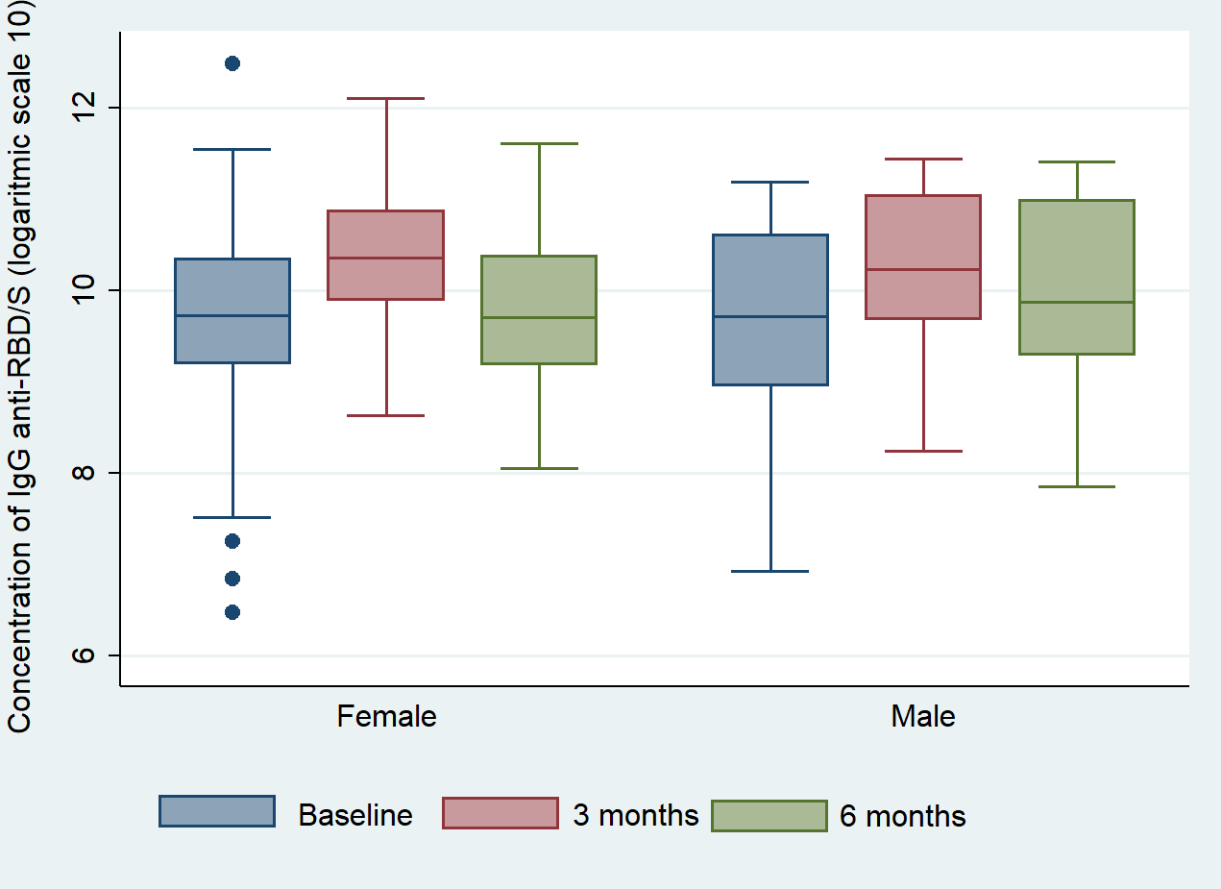


**Supplementary Figure 3.** Anti-RBD/S IgG concentration (logarithmic_10_ scale) reported in the boxplots (and outliers) for participants by smoking status at the three different time points of observation.


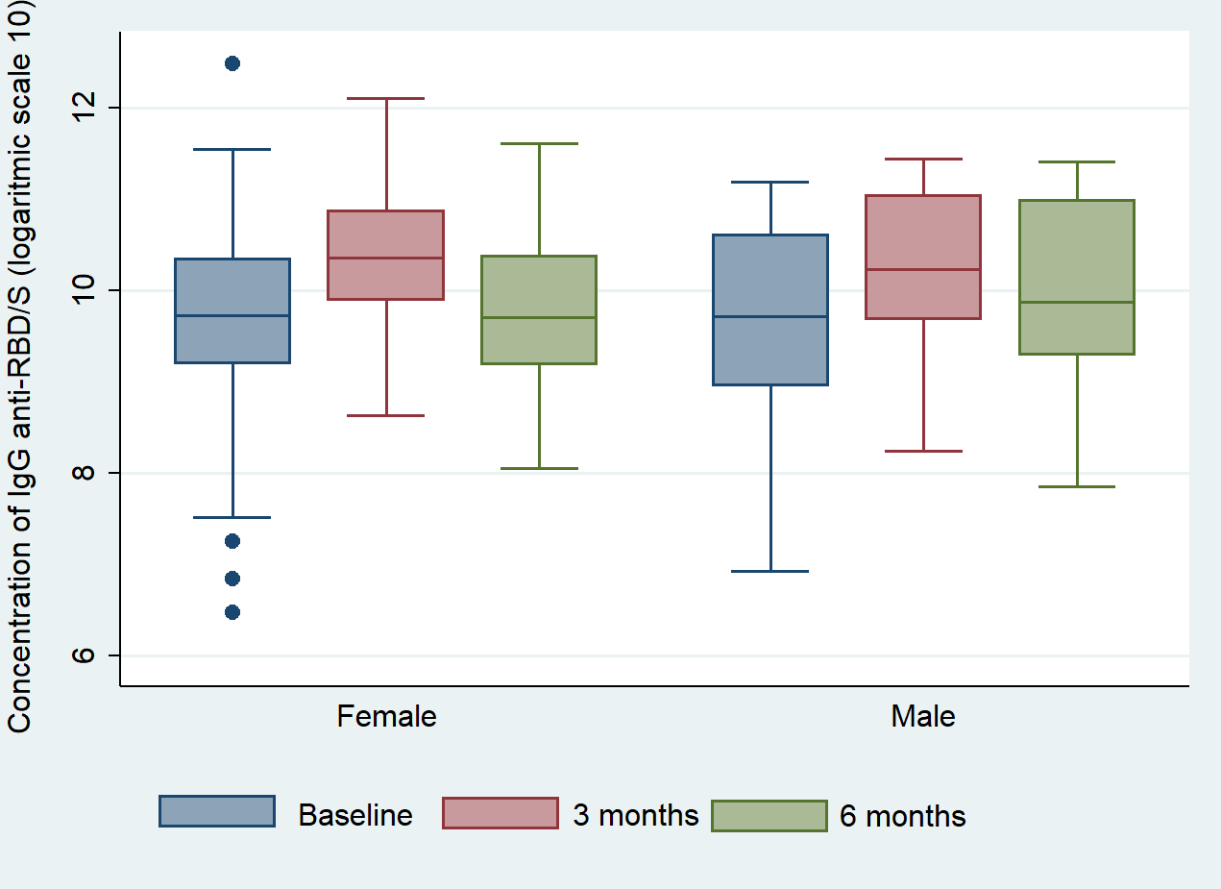

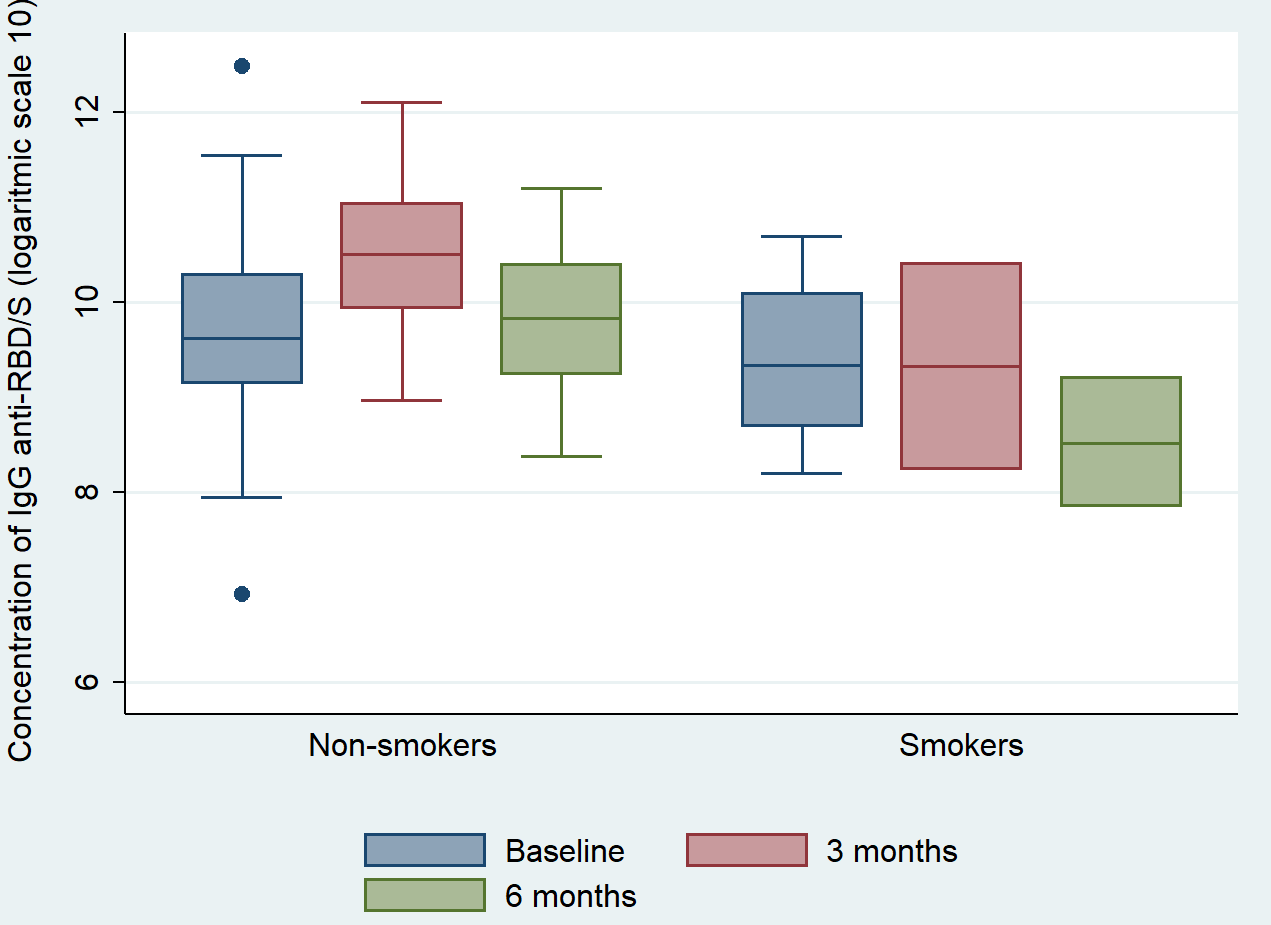


**Supplementary Figure 4.** Anti-RBD/S IgG concentration (logarithmic_10_ scale) reported in the boxplots (and outliers) for participants by chronic condition status at the three different time points of observation.


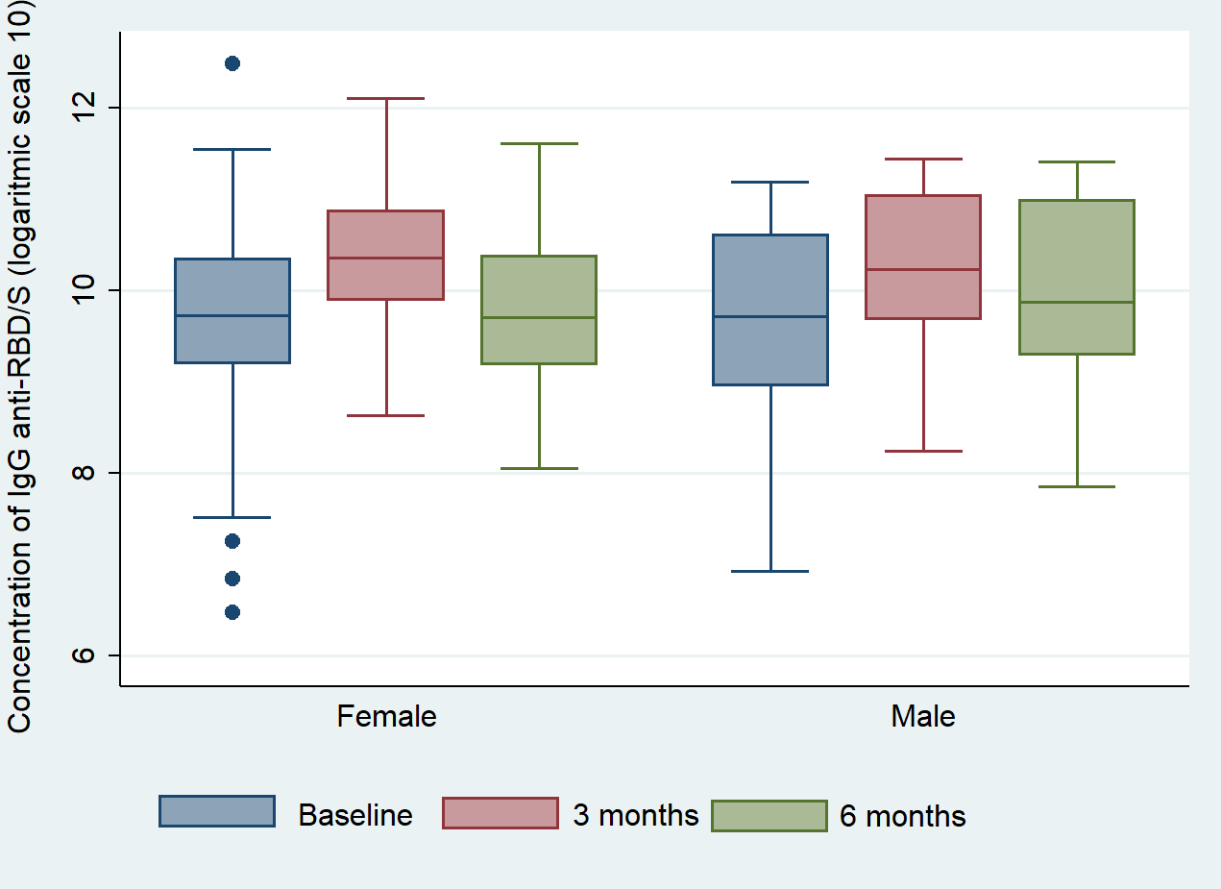

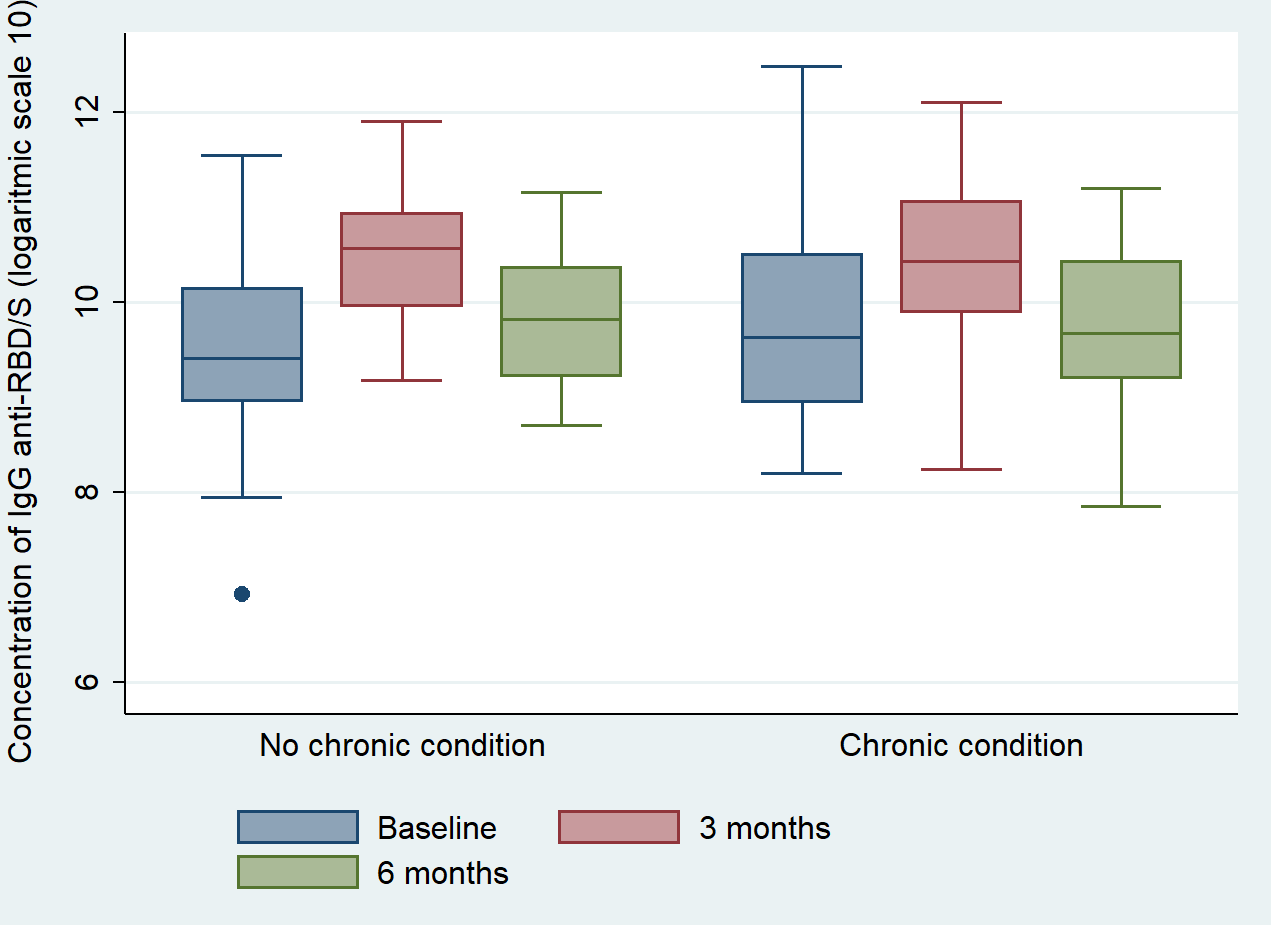

Supplement: Supplementary file 1 — Supplementary Material 1: An additional file is provided (Supplementary data.docx) with: (a) Supplementary Table 1. Comparison of sex and age distribution between all hospital healthcare workers (Hospital HCWs) and those included in the study (Study HCWs); (b) Supplementary Table 2. Comparison between participants and non-participants at 3 months post-COVID-19 vaccination; (c) Supplementary Table 3. Comparison between participants and non-participants at 6 months post-COVID-19 vaccination. (d) Supplementary Figure 1. Anti-RBD/S IgG concentration (logarithmic10 scale) reported in the boxplots (and outliers) for participants by sex at the three different time points of observation; (e) Supplementary Figure 2. Anti-RBD/S IgG concentration (logarithmic10 scale) reported in the boxplots (and outliers) for participants by age group at the three different time points of observation; (f) Supplementary Figure 3. Anti-RBD/S IgG concentration (logarithmic10 scale) reported in the boxplots (and outliers) for participants by smoking status at the three different time points of observation; (g) Supplementary Figure 4. Anti-RBD/S IgG concentration (logarithmic10 scale) reported in the boxplots (and outliers) for participants by chronic condition status at the three different time points of observation [file 12879_2026_13571_MOESM1_ESM.docx]
